# Antibacterial and anti-inflammatory ZIF-8@Rutin nanocomposite as an efficient agent for accelerating infected wound healing

**DOI:** 10.3389/fbioe.2022.1026743

**Published:** 2022-10-05

**Authors:** Xiaomin Xia, Xujun Song, Ying Li, Wenxue Hou, Hanlin Lv, Feng Li, Yanan Li, Jie Liu, Xue Li

**Affiliations:** ^1^ Department of Stomatology, The Affiliated Hospital of Qingdao University, Qingdao University, Qingdao, Shandong, China; ^2^ School of Stomatology, Qingdao University, Qingdao, Shandong, China; ^3^ Dental Digital Medicine and 3D Printing Engineering Laboratory of Qingdao, Qingdao, Shandong, China; ^4^ Dental Biomaterials Technology Innovation Center of Qingdao, Qingdao, Shandong, China

**Keywords:** MOF, Rutin, nanocomposite, antibacterial, antiinflammation

## Abstract

Essentially, wound healing is a complicated physiological process in which there exists an interaction between the organism’s immune regulation and antimicrobial therapy. However, multiple drug-resistant bacteria implicated in chronic non-healing wound are not merely impeding the cure process, but more than a burden on economic and social development. Due to the inefficiency of conventional antibiotics, nanomedicine in the biomedical field is emerging as a prospective anti-infective therapy method. Herein, a novel nano-drug with antibacterial and anti-inflammatory characteristics was synthesized by loading Rutin into zeolitic imidazolate framework-8 (ZIF-8), abided by the principle of electrostatic adsorption. The synthetic ZIF-8 loaded Rutin (ZIF-8@Rutin) was affirmed by testing the changes in the diameter and chemical functional group. Interestingly, the ladened Rutin afforded nanocomposite with anti-inflammatory activity by its antioxidant capacity for the polarization of macrophages. Further, the prepared ZIF-8@Rutin exhibited highly effective antibacterial activity against *Escherichia coli* and *Staphylococcus aureus in vitro*. More importantly, it could shorten the infected wound healing process and alleviate the inflammation around the wound *in vivo*. Also, ZIF-8@Rutin had acceptable cytocompatibility. Thus, ZIF-8@Rutin may become a multifunctional nanomedicine with anti-inflammatory and bactericidal properties to promote infected wound healing.

## 1 Introduction

The wound sounds like a superficial injury to tissue but could be intricate and intractable for an individual’s health. The global wound healthcare burden was assessed at USD 18.4 billion in 2018 and is forecast to grow at 3.9% until 2026 (Sen, 2019; Monika et al., 2022). More seriously, Chronic non-healing wounds are the most tremendous burden to health care and affect over 6.5 million people in US alone (Chen et al., 2020). Chronic non-healing wounds are often characterized by infection or biofilm and aggrandize the production of proinflammatory cytokines. The prolonged inflammation then brings about the inhibition of fibroblast generation, which is imperative for wound remodeling. Although using antibiotics is always a critical measure (Paitan, 2018), the irrational application of antibiotics has produced several side effects (Björnsson, 2017; Shenoy et al., 2019). Regrettably, the emergence of drug-resistant bacteria fear hinders the healing of chronic wounds. Thus, to propose a novel and high-efficiency strategy that equips with dual functionality of sterilization and anti-inflammatory is urgently needed.

With the explosive development of nanotechnology, nanomaterials may represent a promising candidates for the treatment of antibacterial (Vimbela et al., 2018). Metal-Organic Frameworks (MOFs) are coordination polymers with intramolecular pores formed by the self-assembly of organic ligands and metal ions through coordination bonds (Chai et al., 2021). Among these compounds, based on the regular pore structure (Jiang et al., 2018) and the large specific surface area, ZIF-8, which is a dodecahedral coordination compound formed by the ligand 2-methylimidazole and metal ion Zn^2+^ has been widely used in the fields of biomedicine. Numerous studies have shown that ZIF-8 can be used as a reservoir for zinc metal ions expressing efficient bactericidal activity, which includes bactericidal activity in Gram-positive and Gram-negative bacteria (Wan et al., 2021). Because of the remarkable sterilization performance, ZIF-8 could also be an effective drug carrier against drug-resistant bacteria. Such as modifying ZIF-8 encapsulated squaraine to enhance aPDT efficacy against drug-resistant planktonic bacteria and its biofilm (Bagchi et al., 2019). Nevertheless, the components of the organic ligand and metal ions generally produce reactive oxygen species (ROS) to act as bactericidal agents. While the excess ROS could cause inflammation in surrounding healthy tissues and organic ligand is toxicity (Vimbela et al., 2018; Li et al., 2019a; He et al., 2020). Due to the potential inflammatory weakness, which is undesirable for the wound healing process, endowing ZIF-8 with the characteristics of scavenging excess ROS or anti-inflammatory effect during the sterilization process is advantageous.

As a naturally occurring polyphenols, plentiful pieces of literature verified that flavonoids have comprehensive pharmacological functions, covering anti-cancer (Raj et al., 2021), anti-inflammatory (Arowoogun et al., 2020) and anti-viral. Significantly, the latest study reported that quercetin and Rutin could be active components in the inhibition of SARS-CoV-2 through the JAK-STAT signaling pathway to kill the Coronavirus Disease 2019 (COVID-19) (Huang et al., 2020). The antioxidant activity of Rutin has attracted the most attention, even related studies support the diet supplemented with (Enogieru et al., 2018; Wani et al., 2021). However, due to the extremely poor solubility (Mauludin et al., 2008), Rutin is difficult to act directly on the organism.

**SCHEME 1:**
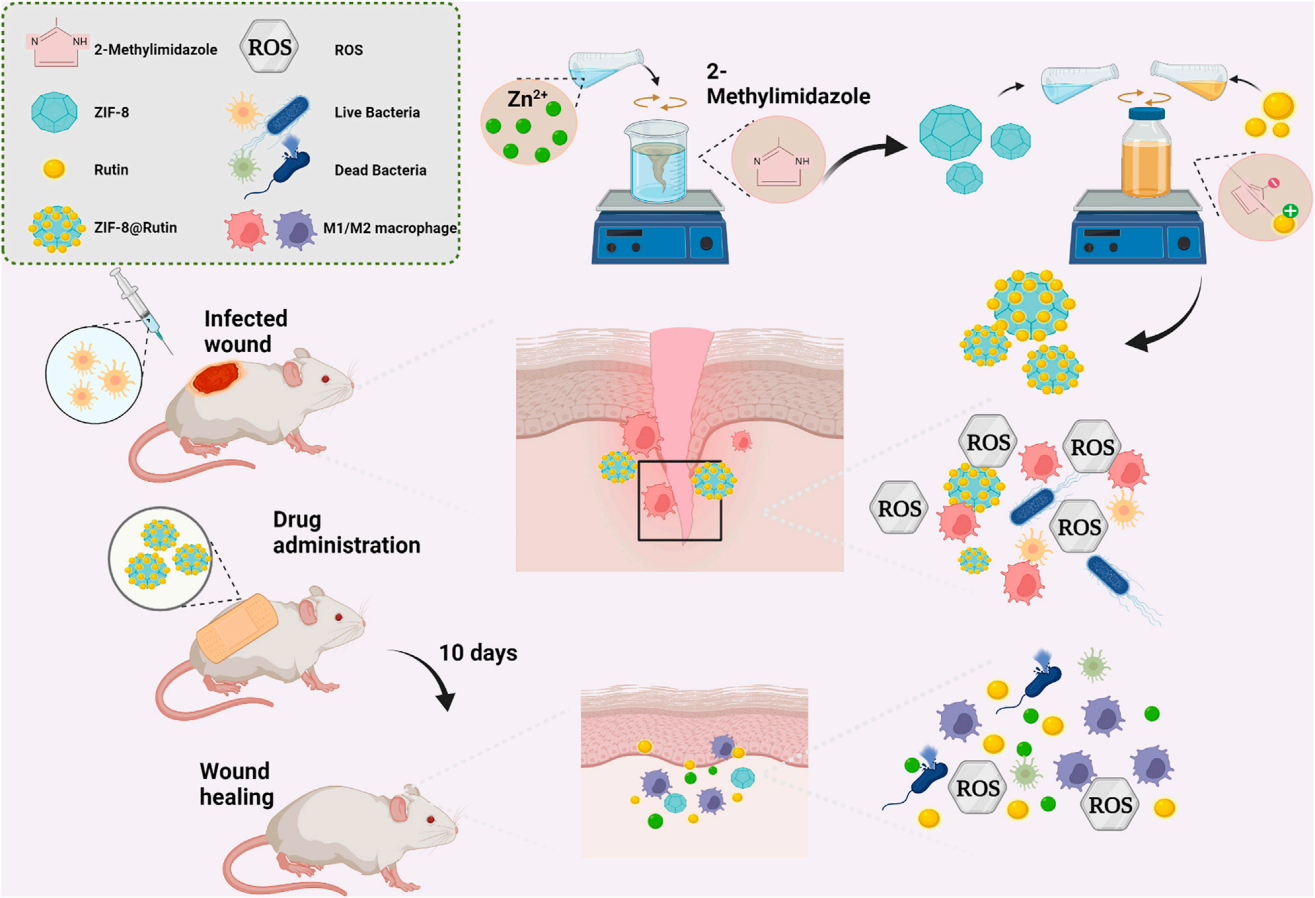
Schematic representation of ZIF-8@Rutin nanocomposite acting synergistically on an infected wound by eliminating bacteria and modulating the inflammatory response to promote wound healing. ZIF-8@Rutin NPs undergo structural collapse and hydrolysis when they enter body fluids, releasing free Zn^2+^, which confers superior antibacterial properties to the NPs. In parallel, bacteria or free Zn^2+^ can exacerbate local inflammation by producing large amounts of ROS. Interestingly, the enzyme-mimicking activity of the Rutin could remove the excess ROS from the ZIF-8 framework, thereby significantly shortening the inflammatory process. Furthermore, doped Rutin protects cells from oxidative stress, depending on the ability to scavenge ROS. During the anti-inflammatory activity, ZIF-8@Rutin also has the capacity to polarize macrophages M1 to an anti-inflammatory M2 phenotype. ZIF-8@Rutin nanocomposites possess promising therapeutic potential for accelerating the healing of infected wounds by exploiting their superior antibacterial and anti-inflammatory properties.

In the present study, as shown in Scheme 1, ZIF-8 loaded with Rutin (ZIF-8@Rutin) was synthesized abiding by the principle of electrostatic adsorption. A novel ZIF-8@Rutin nanocomposite with both anti-inflammatory and antibacterial functions was announced. Rutin modification can make up for the lack of anti-inflammatory activity of ZIF-8 and improve the biosafety of nanocomposites. Our new findings may pave the way for wound healing and potent bactericidal activity against drug-resistant bacteria. Therefore, the purpose of this study is to: (1) design and characterize ZIF-8@Rutin nanoparticles. (2) evaluate the effect of ZIF-8@Rutin on inhibiting the secretion of inflammatory factors and promoting the polarization of anti-inflammatory M2 macrophages. (3) test the *in vitro* and *in vivo* antibacterial activity of ZIF-8@Rutin against *Escherichia coli* and *Staphylococcus aureus* local infection. The experimental results confirmed our hypothesis: (1) the cytotoxicity of ZIF-8NPs was reduced by order of magnitude by Rutin loading; (2) ZIF-8@Rutin have strong antibacterial properties both *in vitro* and *in vivo*; (3) the addition of Rutin could alleviate the inflammatory response and promote the healing of infected wounds.

## 2 Material and methods

### 2.1 Materials and reagents

No further purification was required as all chemicals were obtained from commercial suppliers. Zn (NO_3_) _2_^.^6H_2_O and 2-Methylimidazole were obtained from Sigma (Aldrich, St. Louis, MO, United States). RIZOL was purchased acquired from Life Technologies (Carlsbad, CA, United States). Takara (Shiga, Japan) supply RNA-iso Plus, Prime Script RT reagent kit and TB Green Premix Ex Taq II kit. Kits for the detection of superoxide dismutase (SOD), catalase (CAT) and total antioxidant volume (T-AOC) were purchased from Solarbio (Beijing, China). In addition to human gingival fibroblasts (HGFs, ScienCell, SanDiego, CA, United States), murine macrophages (RAW 264.7 cell line), *Escherichia coli* (*E. coli*) ATCC 25922 and *Staphylococcus aureus* (*S. aureus*) ATCC 29213 were obtained from American Type Culture Collection (ATCC, Manassas, VA). Rutin hydrate, Calcian-AM/PI Double Staining Kit and all chemical reagents and other biological kits were obtained from Dalian Meilun Biotechnologies Co., Ltd. No additional purification of all reagents used.

### 2.2 Preparation of ZIF-8 and ZIF-8@Rutin NPs

ZIF-8 NPs were prepared according to the previously reported method. In brief, 0.74 g (2.5 mmol) of Zn (NO_3_)_2_^.^6H_2_O and 3.28 g (40 mmol) were dissolved in 20 mL and 80 ml of methanol respectively and fully dissolve. After vigorously stirring the Zn (NO_3_) _2_^.^6H_2_O solution and the 2-methylimidazole solution for 4 h, the mixture was stirred at 50 °C for 1 h and then immediately centrifuged at 9,500 rpm for 15 min. The crude product was scrubbed three times with methanol and dried at 60 °C for 12h, and ground resulting in a white solid powder of ZIF-8. Regarding ZIF-8@Rutin NPs, equal amounts of Rutin and ZIF-8 powder were dissolved in methanol respectively, and then the two solutions were mixed and sealed in a silk-top bottle with stirring 24 h at room temperature. The prepared hybrid nanoparticles were centrifuged after 24 h of stirring at room temperature and then collected from the solution. On top of centrifugation, the precipitate was washed three times with methanol, dried and ground into powder in a vacuum dryer to obtain ZIF-8@Rutin powder.

### 2.3 Characterization

The surface morphologies of nanoparticles were observed by transmission electron microscopy (TEM, JEM-2010, JEOL, Tokyo, Japan) and scanning electron microscopy (SEM, Hitachi S-4800, Tokyo, Japan). Particle size analysis of nanoparticles was performed by calculating SEM images (at least 50 molecules at random from the SEM images for each sample) using ImageJ software. The analysis of the internal structure of NPs was conducted by X-ray diffractometry (XRD, Rigaku D-MAX 2500/PC), and the program was set up with parameters consistent with those which were previously reported in the literature (Li et al., 2019a), in comparison with standard XRD patterns.

The release behavior of Zn ions was monitored at 2, 4, 8, 12, 24 h respectively, by using the ICP-OES (Agilent 5,110, America). NPs (160 μg) were added to 4 ml PBS. It was sealed tightly and then left for 24 h at 37 °C in a cell incubator to macerate. 0.5 ml of the supernatant and an equal volume of PBS was pipetted into the flask over a specific period. The collected supernatant was adjusted to 10 ml, diluted 200 times and tested on the machine. All ICP measurements for each set of samples were performed in triplicate.

Dissolve Rutin in methanol to prepare reference solutions at levels of 10, 20, 30, 40, and 50 μg ml^−1^, sonicating for 30 min, read them and plot the calibration curve of the standard stock solution of Rutin with HPLC (Primaide system). The specific parameters were set as follows: the specific flow rate was 0.7 ml min^−1^, the wavelength of the UV detector was adjusted to 257 nm, methanol:0.2% acetic acid, 1:1 (v/v) as the mobile phase, and the column temperature was 37°C. The ZIF-8@Rutin PBS solution was prepared with a concentration of 40 μg ml^−1^ and divided into six parts after it was mixed. The supernatant was drawn at 0, 2, 4, 8, 12, 24 h at 37°C to determine the Rutin content. The data from ICP and HPLC were measured three times for each sample.

### 2.4 SOD, CAT and T-AOC enzyme mimic activity of ZIF-8@Rutin NPs

To assess the antioxidant levels of ZIF-8, Rutin and ZIF-8@Rutin, superoxide dismutase (SOD), catalase (CAT) and total antioxidant capacity (T-AOC) activities were measured with an enzymatic calibrator (Elx800, Bio Tek, United States) based on the recommendations of the manufacturer.

A metallic enzyme, SOD is commonly available in living organisms and is a key scavenger of oxygen radicals. It facilitates the production of H_2_O_2_ and O_2_ by disproportionation of superoxide anions. In the metabolic reaction, superoxide anion (O_2_-) is generated through xanthine and xanthine oxidase reaction system (Weydert and Cullen, 2010). O_2_- can reduce nitro blue tetrazole to generate blue formazan and SOD can remove O_2_- cause inhibiting the production of formazan. SOD enzyme activity was reflected by measuring the content of formazan at UV light 560_nm_. The amount of SOD activity in one unit was defined as a 50% reduction in the amount of enzyme formed by WST-8 toluene burdock.

CAT is the predominant H_2_O_2_ scavenging enzyme and it is able to decompose H_2_O_2_ (Luna et al., 2005). After the CAT working medium is mixed with the test sample according to the manual instruction, the hydrogen peroxide content at UV light 240 nm after 1min was immediately determined at room temperature. The content of hydrogen peroxide is inversely proportional to the CAT enzyme activity.

The total antioxidant level of various antioxidant substances and antioxidant enzymes in the object was determined. The ability to reduce Fe^3+^-tiracizine (Fe^3+^-TPTZ) to blue Fe^2+^-TPTZ in acidic environments reflects the total antioxidant capacity. The SOD, CAT and T-AOC tests were performed, respectively three times.

### 2.5 Cytocompatibility assay for ZIF-8@Rutin NPs

To evaluate the cell compatibility of ZIF-8@Rutin, HGFs testing was performed using a cell counting kit (CCK-8). The fibroblasts were cultured in the well-prepared media for 24 h, 48 h and 72 h. After incubation in 37 °C mediums for 24 h, live cells were determined by CCK-8 using a microplate reader at OD_450nm_ (Bio-Tek, Winooski, VT, United States). Six wells were available in each experimental group. The morphology of HGFs was examined after culturing with different concentrations (10 μg ml^−1^–100 μg ml^−1^) of ZIF-8, Rutin and ZIF-8@Rutin. Briefly, after co-culture were washed three times with PBS, Subsequently, calcian-AM and PI were added to dye the live and dead cells for 30 min. Morphology of cells was using confocal laser scanning microscopy (CLSM, C2si, Nikon, Japan).

### 2.6 Bacterial cultivation and single species biofilm formation

Approval for the use of two bacterial pathogens (*Escherichia coli* and *Staphylococcus aureus*) was obtained from the Institutional Review Board of the Qingdao University School of Dentistry. *E. coli* was cultured in LB broth, meanwhile *S. aureus* was cultured in TSB broth.

#### 2.6.1 Metabolic activity

The bacterial growth activity was determined by co-culturing the bacterial suspension with ZIF-8 (30 μg ml^−1^)、Rutin (50 μg ml^−1^)and ZIF-8@Rutin (40 μg ml^−1^), and the antibacterial property of the materials was evaluated. Specific steps are as follows: a fresh colony is selected and it incubated on a shaker at a speed of 200 rpm at 37°C overnight. Dilute the obtained bacterial suspension with bacterial medium and bacterial medium containing ZIF-8 (30 μg ml^−1^)、Rutin (50 μg ml^−1^) and ZIF-8@Rutin (40 μg ml^−1^) to an OD630 nm value of 0.08, the resulting solution was transfer to a 24-well plate and left to incubate under anaerobic conditions (80% N2, 10% H2 and 10% CO2) for 24 h. Bacterial suspensions were pipetted into a 96-well plate to determine at OD630 nm at 0 h, 2 h, 4h, 8 h, 12 h, and 24 h by a microplate reader (Spectra ax M5, Molecular Devices, Sunnyvale, CA, United States) after coculture.

#### 2.6.2 CFU counts of periodontal biofilm on cover glasses

*E. coli* and *S. aureus* colonies were selected from the fresh bacterial library mixed in pre-configured LB/TSB medium and grown under on a shaker at a speed of 200 rpm at 37°C for 24 h. Extract 1 ml from the above turbid solution and it was diluted with ZIF-8 (30 μg ml^−1^), Rutin (40 μg ml^−1^) and ZIF-8@Rutin (40 μg ml^−1^) to an OD630 nm value of 0.04, and put into coculture for 24 h under the anaerobic condition (80% N2, 10% H2 and 10% CO_2_). The bacterial solution after co-cultivation with ZIF-8 (30 μg ml^−1^), Rutin (40 μg ml^−1^)and ZIF-8@Rutin (40 μg ml^−1^)was diluted 10–8 times with PBS, and 20 μL of the above bacterial liquid was evenly spread on the agar plates and incubated in 37°C incubator for 24 h.

#### 2.6.3 TEM inspection on the bacterial morphology

Taking the co-cultivation of *E. coli* with ZIF-8@Rutin as an example, the method of co-cultivation of materials is the same as the previously described one. 0.5 ml of the bacterial suspension is aspirated and centrifuged at 6,000 rpm for 15 min at room temperature. The obtained pellet was washed three times with sterile PBS and fixed with 2.5% glutaraldehyde for 2 h. The samples were sprayed with gold, and then they were observed by transmission electron microscopy.

### 2.7 Expression of M1/M2 phenotype macrophage-related cytokines

Anti-inflammatory properties displayed by ZIF-8@Rutin NPs were evaluated with mouse macrophages (RAW 264.7) as an *in vitro* model of inflammation. The culture conditions were the same as HGFs which were not included in antibiotics. The concentration of RAW cells was controlled at 1 × 10 ^6^ cells per mL and cells were stimulated by *P. gingivitis*-LPS (1 μg ml^−1^) for 24 h, which were incubated in 6-well plates with ZIF-8 (30 μg ml^−1^), Rutin (40 μg ml^−1^) and ZIF-8@Rutin (40 μg ml^−1^) for 24 h.

The absence of NPs-treated LPS-stimulated cells served as a negative control. Targeted M1 phenotype macrophage-associated cytokines (IL-1β, IL-6, and TNF-α) and M2 phenotypic macrophage-associated cytokines (IL-10, Arg-1, and TGF-β) were quantified by using the TB Green Premix Ex Taq™ II.

A 2^ΔΔCT^ assay was performed to examine relative gene expression and standardize the data to the housekeeping gene β-actin. The entire experiment was repeated in triplicate. The sequences of the extracts used for the qPCR assay are shown in Table 1.

**TABLE 1:** Primer sequences used in this study.

Gene	Forward sequence (5 ′ to 3 ′)	Reverse sequence (50 ′ to 30 ′)
β-Actin	CAT​CCG​TAA​AGA​CCT​CTA​GCC​AAC	ATG​GAG​CCA​CCG​ATC​CAC​A
IL-1β	TCC​AGG​ATG​AGG​ACA​TGA​GCA​C	GAA​CGT​CAC​ACA​CCA​GCA​GGT​TA
IL-6	CCA​CTT​CAC​AAG​TCG​GAG​GCT​TA	CCA​GTT​TGG​TAG​CAT​CCA​TCA​TTT​C
TNF-α	ACT​CCA​GGC​GGT​GCC​TAT​GT	GTG​AGG​GTC​TGG​GCC​ATA​GAA
TGF-β	CTT​CAG​CCT​CCA​CAG​AGA​AGA​ACT	TGT​GTC​CAG​GCT​CCA​AAT​ATA​G
Arg-1	TGT​GTC​CAG​GCT​CCA​AAT​ATA​G	AGC​AGG​TAG​CTG​AAG​GTC​TC
IL-10	ATG​CTG​CCT​GCT​CTT​ACT​GAC​TG	CCC​AAG​TAA​CCC​TTA​AAG​TCC​TGC

### 2.8 Establishment of *in vitro* inflammation model

BALC/c male mice (18–22g, 6-8w) were obtained from Jinan Pengyue laboratory animal breeding co. ltd. All experiments are carried out in accordance with the Institutional Animal Care and Use Committee of Qingdao University, following the instructions.

The model of inflammation *in vitro* was established by dropping 100 μL of *S. aureus* suspension (1 × 10^6^ CFU ml^−1^) on a full-thickness circular wound with a diameter of 5 mm on the back of mice. The back wounds of mice were dripped with PBS as a negative control (*n* = 5). After the inflammation model was successfully established, the infected wounds were deal with dressings comprising PBS, ZIF-8, Rutin and ZIF-8@Rutin to seal the wounds, respectively. The dressing was changed every 2 days, and the course of treatment lasted 10 days. The healing area of the back wounds of the mice in different experimental groups was recorded on 0, 2, 4, 6, 8, 10 days after the inflammation model was established. Finally, all tissues around the wound on the back of the mouse were excised on the 10th day. Tissue sections were fixed with 10% formalin and stained with H&E and Masson, respectively. The results of H&E staining and Masson staining will demonstrate the histological characteristics of wound healing in the different treatment groups and the regeneration of the wound will be assessed by observing the re-epithelialization of the tissue, the infiltration of inflammatory cells and the deposition of collagen fibers. As for the mechanism of ZIF-8@Rutin’s anti-inflammatory effect, we quantified the expression of inflammation-related cytokines in skin tissue by immunohistochemical staining and immunofluorescence staining. TNF-α was chosen as a typical pro-inflammatory factor to determine the anti-inflammatory capacity of the nanocomposites *in vivo*, alongside Arg-1 as a typical anti-inflammatory factor.

### 2.9 Statistical analysis

All experiments were repeated at least three times. Statistical analysis was performed by using GraphPad software. Significant effects of variables were detected using one-way or two-way ANOVA to compare the means of groups at *p*-values less than 0.05.

## 3 Results and discussion

### 3.1 Characterization of ZIF-8 NPs and ZIF-8@Rutin NPs

**FIGURE 1:**
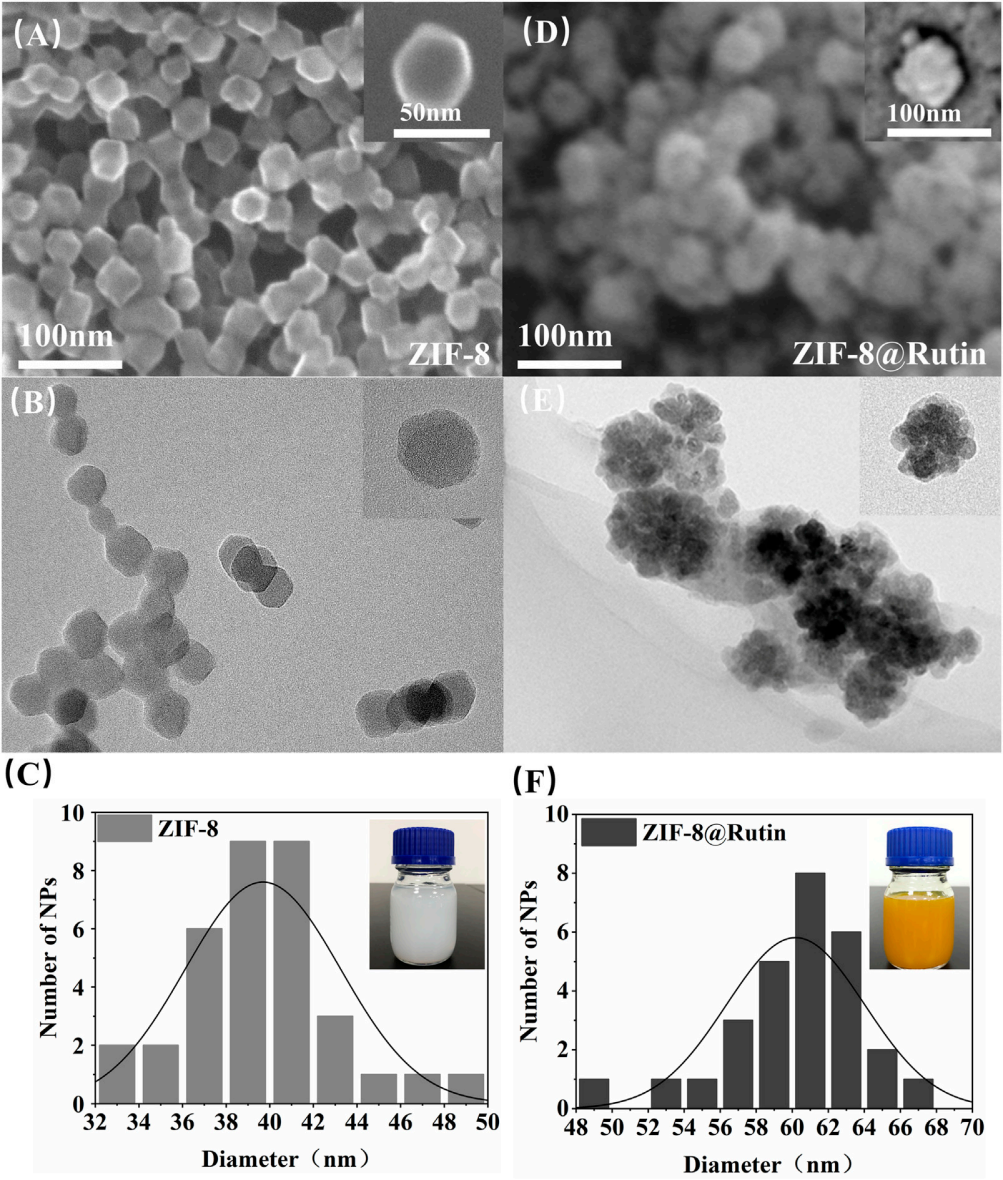
The morphology and size profiles of ZIF-8 and ZIF-8@Rutin nanocomposites. Representative SEM images of ZIF-8**(A)**, ZIF-8@Rutin**(D)** and TEM images of ZIF-8**(B)**, ZIF-8@Rutin**(E)**. The insets in **(A,B,D,E)** represented individual magnified electron microscopy images of the corresponding nanocomposites. The particle size distribution of the nanocomposites showed a homogeneous size concentration of ZIF-8**(C)** and ZIF-8@Rutin NPs**(F)**. The illustrations in **(C,F)** showed the variation in color of the different liquid synthesis systems for NPs gradually from white to yellow.

As shown in the SEM and TEM images of ZIF-8 and ZIF-8@Rutin (Figures 1A,B), all the nanoparticles have a regular dodecahedral morphology with a uniform particle size distribution. The diameter of the ZIF-8 NPs was about 40 nm (Figure 1C). Interestingly, from the images of Figures 1D,E, the synthesized ZIF-8@Rutin NPs have a “Flower”-like morphology, and the particle diameter was significantly more extensive than that of the ZIF-8 single (Figure 1F). Through the above test results, we could confirm the successful loading of Rutin into ZIF-8. As for the binding mechanism, we speculated that it was under the action of electrostatic adsorption. The study raised momentous electrostatic flexibility of the ZIF-8 framework; C−H groups and metal cations have positive evoked potential on the surface of nanoparticles (Novaković et al., 2015; Wu et al., 2021). The positively charged ZIF-8 surface could be attracted to the negatively charged hydroxyl group of Rutin (the functional groups of −OH offer reactive sites), and the aggregation between the two occurred (Sentkowska et al., 2013). Moreover, the color of ZIF-8@Rutin dispersion changed from milky white to yellow (diagram on delivery card), which also illustrates the successful launch of conventional food supplements.

**FIGURE 2:**
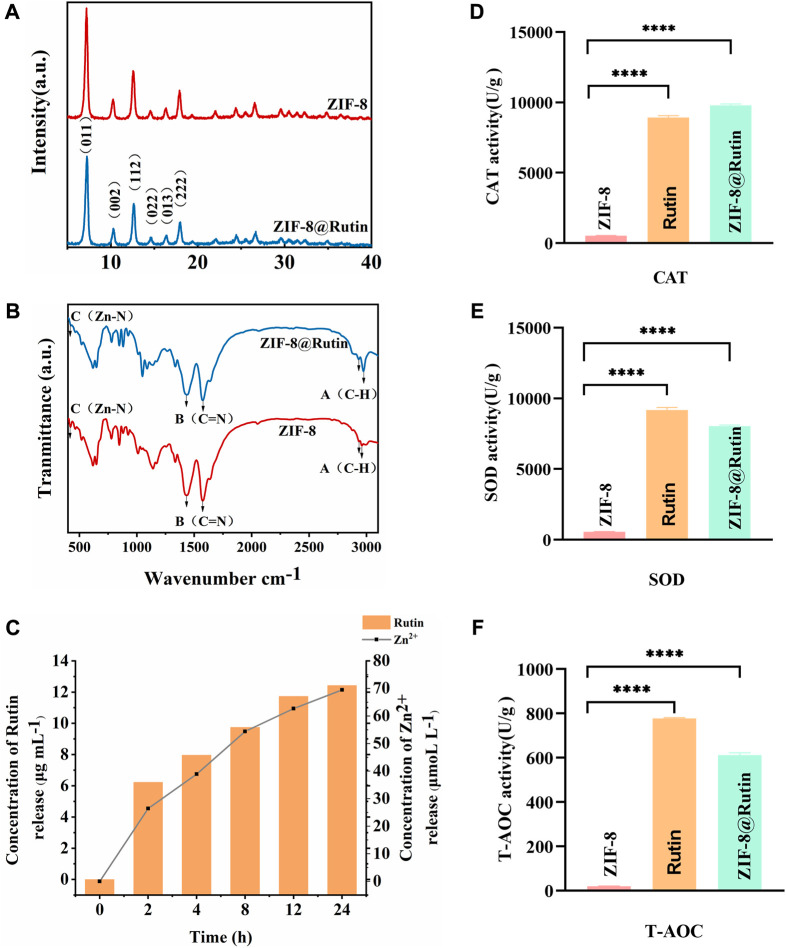
Characterization of ZIF-8 and ZIF-8@Rutin nanocomposites. **(A)** XRD analysis of ZIF-8 and ZIF-8@Rutin. **(B)** Infrared spectroscopy (FTIR) of ZIF-8 and ZIF-8@Rutin NPs. **(C)** Release behaviors of zinc ions and Rutin in ZIF-8@Rutin NPs PBS solutions for 24 h **(D–F)** Quantitative analysis of the enzymatic activities of ZIF-8 and ZIF-8@Rutin. Significant differences between the groups were indicated by different letters (n = 3, ****: *p* < 0.0001).

The structural characteristics of ZIF-8 and ZIF-8@Rutin NPs were examined by XRD and FTIR analysis. As shown in Figure 2A, the (011), (002), (112), (022), (013), and (222) diffractions of ZIF-8 NPs all have six sharp diffraction peaks, indicating that the sample has a high degree of crystallinity (Park et al., 2006). However, The XRD peak height of ZIF-8@Rutin NPs has an inevitable decrease, which may be due to the lower crystal crystallization after loading with Rutin than that of ZIF-8 alone. As shown in Figure 2B, the infrared absorption peaks at 2943.80 cm^−1^ and 2958.68 cm^−1^ (point A) belong to the expansion and contraction peaks of the C−H bond of methyl and imidazole, respectively (Remanan and Zhu, 2020). In addition, an inevitable at 2943.80 cm^−1^ and 2958.68 cm^−1^ corresponding to the vibration of C−H increased in ZIF-8@Rutin NPs. At 1429.42 cm^−1^ (point B), the stretching vibration peak of the C=N bond on the imidazole ring appeared, and at 426. cm^−1^ (point C), the stretching vibration peak of Zn-N appeared (Anisi et al., 2021). The absorption bands at 1204 cm^−1^ and 1043 cm^−1^ were associated with the stretching vibrations of the C–O–C groups in the ZIF-8@Rutin, while ZIF-8 was absent (Remanan and Zhu, 2020). Overall, FTIR analysis confirmed the physical interaction between Rutin and ZIF-8 and the successful loading of Rutin. The XRD pattern and FTIR results coincided with the previously reported structural data, indicating that the structure of the original ZIF-8 was not changed after loading Rutin.

It is worth noting that one of the advantages of nano-drug delivery systems is sustainability release. Sustained release could keep steady concentration in local and maintain the condition in a stable state. Thus, in the present study, Rutin and Zn^2+^ cumulative release experiments were implemented by high-performance liquid chromatography (HPLC). As shown in Figure 2C, during the first 2 hours, the concentration of Rutin released could reach half of the final release concentration, which was probably due to the instantaneous collapse of the structure of ZIF-8 itself as a result of the hydrolysis (Shearier et al., 2016). At 24 h, the attention of Rutin released has essentially reached equilibrium. The release showed a slow upward trend within 24 h, and the prolongation of Rutin release can be considered as an attraction between positively charged metal ions and negatively charged hydroxyl groups (-OH).

Rutin is a plant extract with excellent antioxidant and other pharmacological activities, but has no apparent cytotoxicity (Cushnie and Lamb, 2005). There are two main mechanisms by which Rutin has antioxidant activity. Studies have shown that flavonoids react with free radicals and block free radical chain reactions (Benavente-García et al., 1997). Another mechanism is the relationship between the structure of flavonoids and free radical trapping. The 0-dihydroxy (catechol) structure in the β-ring combines with the 2,3-double bond of the 4-oxo function, both of which are involved in electronic dislocations; and 3- (a) -and 5- (b) -hydroxyl groups with maximum radial clearance and the most substantial radical absorption (Benavente-García et al., 1997). The multiple methylations of the hydroxyl substituents enable the flavonoids to possess favorable anti-lipid peroxidation activity, while inhibiting the mitochondrial membrane permeability transition, thus increasing the pharmacological potential of these compounds for use in antioxidant therapy (Of et al., 1998). As shown in Figures 2D,F, ZIF-8@Rutin displayed decent peroxidase activity and superoxide dismutase activity. Other splendid research also affirmed that among flavonoids, Rutin exhibits the highest free radical scavenging capability because of its ability to scavenge free radicals DPPH ^***•***^, which improved the total antioxidant capacity (Material and Material, 2005; Londero et al., 2021). Then, in ZIF-8@Rutin NPs, Rutin can neutralize the excess ROS produced by ZIF-8 NPs and avoid damage to healthy tissues. Meanwhile, there is a clear and significant difference between the Rutin and ZIF-8@Rutin. The above results indicate that the presence of ZIF-8 can provide a large amount of reactive oxygen species, which further provides experimental support for the hypothesis that ZIF-8@Rutin NPs utilize a large amount of reactive oxygen species for sterilization in this experiment.

### 3.2 Cytotoxicity of Rutin, ZIF-8 NPs, and ZIF-8@Rutin NPs

As a novel biomedical material, biosafety evaluation is essential before clinical application. Previous experiments reported that ZIF-8 was unstable to release Zn^2+^ due to the hydrolysis in aqueous solution (Shearier et al., 2016) and the free metal ions made MOFs toxic ones (Vimbela et al., 2018). Despite the cytotoxic peculiarity of free Zn^2+^, the lack of it will cause the reduction of cytokines and reactive oxygen species, which will have a specific impact on the body’s defense against pathogenic microorganisms (Rath et al., 2016). Zn^2+^ has been reported to play an important role in wound healing: Zn^2+^-deficient wounds typically experience slower healing times (Glutsch et al., 2019). Therefore, it is required to control Zn^2+^ concentration at a safe level.

**FIGURE 3:**
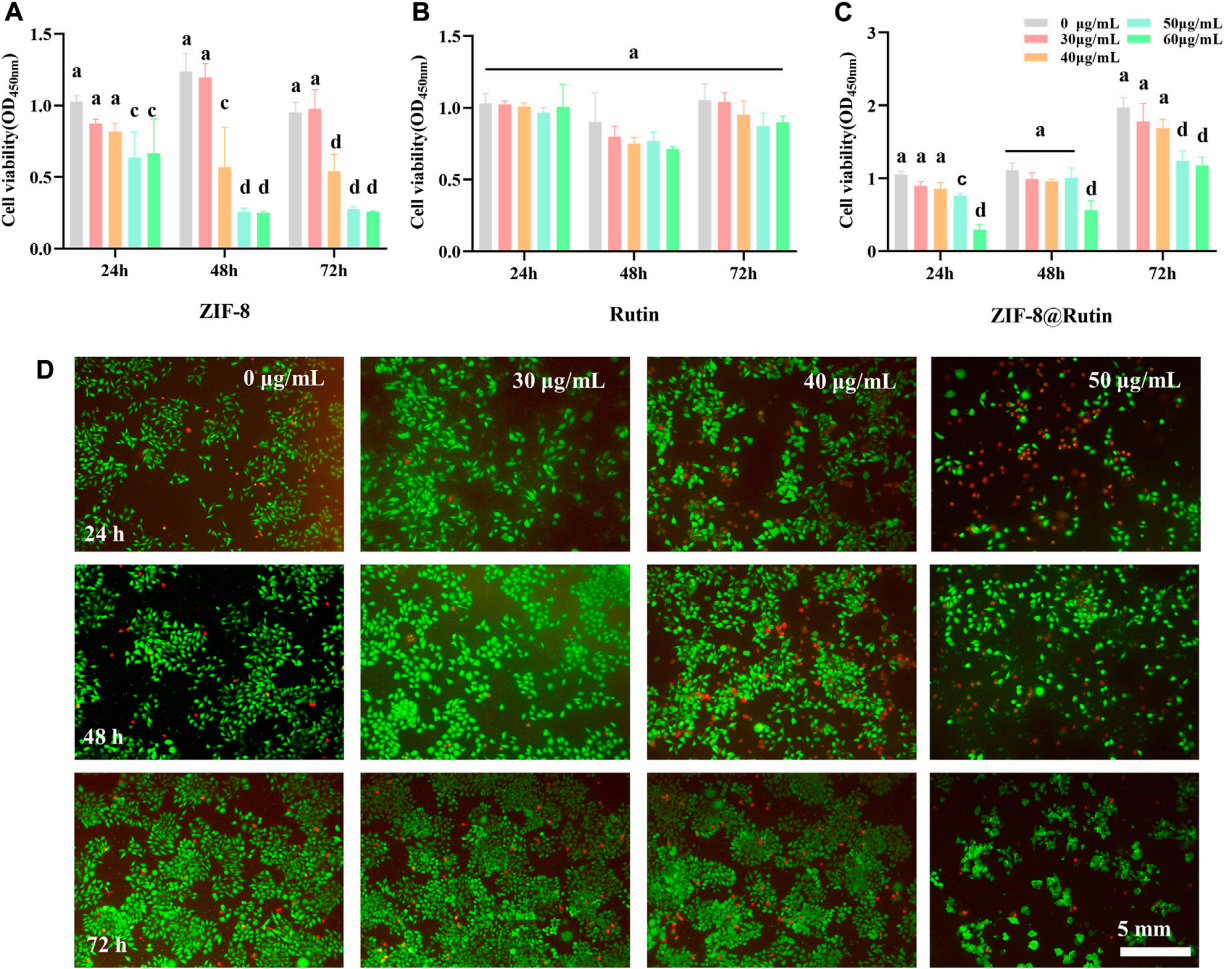
Cytotoxicity of ZIF-8, Rutin and ZIF-8@Rutin nanocomposites. **(A–C)** Vigor of HGFs incubated with ZIF-8, Rutin and ZIF-8@Rutin NPs (0, 30, 40, 50, 60 μg ml^−1^) for 24 h, 48 h, and 72 h respectively. A 96-well plate without NPs (0 μg ml^−1^) was used as a blank control. The solid line represents whether the experimental group is statistically different compared to the blank control. **(D)** Representative images of HGF cultured with 30 μg ml^−1^, 40 μg ml^−1^, and 50 μg ml^−1^ of ZIF-8@Rutin NPs. Live cells were represented in green; dead cells were in red. Dissimilar letters indicated values that were significantly different from each other group (*n* = 6, *p* < 0.05).

In this experiment, the cytotoxicity of ZIF-8 NPs, Rutin and ZIF-8@Rutin NPs was assessed using CCK-8 assay and calcian-AM/PI cell staining. The CCK-8 experiment results (Figures 3A–C) showed that the ZIF-8 NPs solution appeared deleterious when the concentration was higher than 30 μg ml^−1^. Significant cytotoxicity was observed in ZIF-8@Rutin NPs solution when the concentration was higher than 40 μg ml^−1^, while the Rutin solution did not show cytotoxicity, even the engagement was over 60 μg ml^−1^. Meanwhile, we have gained similar results from the fluorescent staining of live and dead cells (Figure 3D). It is gratifying that the toxicity of ZIF-8@Rutin NPs was sharply reduced compared to ZIF-8 NPs at the same concentration. Rutin has been shown to possess significant antioxidant activity in Figures 2D–F and intrinsically low toxicity (Figure 3B). These could fully explain the significant reduction in cytotoxicity of ZIF-8 NPs after loading with Rutin.

### 3.3 Antimicrobial properties of the ZIF-8@Rutin NPs

**FIGURE 4:**
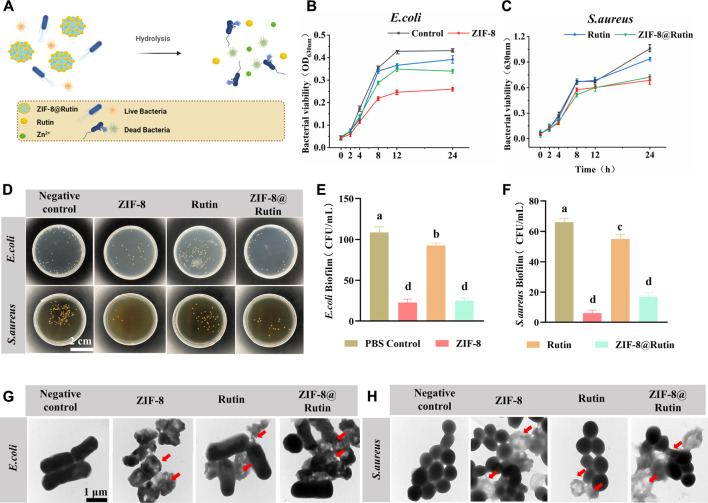
Antimicrobial properties of ZIF-8, Rutin and ZIF-8@Rutin nanocomposites. **(A)** Antimicrobial schematic of ZIF-8@Rutin NPs. **(B,C)** Inhibitory behavior of ZIF-8 (30 μg ml^−1^), Rutin (40 μg ml^−1^) and ZIF-8@Rutin (40 μg ml^−1^) on the metabolic activity of *E. coli* **(B)** and *S. aureus* **(C)** within 24 h. **(D)** Representative pictures of *E. coli* and *S. aureus* colonies on agar plates after different groups of treatments. **(E,F)** Statistical graphs of CFU counts of single-species biofilms of *E. coli* **(E)** and *S. aureus* **(F)**. Representative TEM **(G,H)** images of *E. coli* and *S. aureus*. The morphology of the bacteria was altered at the red arrows. Dissimilar letters indicated values that were significantly different from each other group (*n* = 6, *p* < 0.05).

Figure 4A is a brief diagram of the sterilization principle of ZIF-8@Rutin NPs, namely, the ZIF-8@Rutin releases metal ions and Rutin through the collapse of the structure. To confirm the antibacterial properties of ZIF-8@Rutin NPs, the viability of the bacteria was determined in this study by co-culture. The significant defence is the skin, which consists of the body’s significant barrier against external aggressions (Pullar et al., 2017). The micro-ecological balance system of the skin usually incorporates common microorganisms such as *Staphylococcus*, *Corynebacterium* and *Propionibacterium* (Holland and Bojar, 2002). When the stable is disrupted by pathogenic bacteria, wound infections can occur. The bacteria associated with wound infections are mainly Gram-positive bacteria, of which *Staphylococcus aureus* (*S. aureus*) is particularly pathogenic (Bessa et al., 2013). *Escherichia coli* (*E. coli*), a crucial representative of Gram-negative, has been reported with alto-frequency among antibiotic-resistant strains recently (Paitan, 2018). Consequently, *S. aureus* and *E. coli* were chosen as representative pathogens for this antimicrobial assay.

Representative growth curves of *E. coli* and *S. aureus* were shown in Figures 4B,C. For both pathogens, ZIF-8 NPs and ZIF-8@Rutin NPs groups revealed significant growth inhibitory behavior compared with the control group. Interestingly, Rutin also displayed a weak tendency to inhibit the growth of *E. coli* than *S. aureus*, probably because *E. coli* is more susceptible to Rutin concerning *S. aureus* (Amin et al., 2015; Wang et al., 2021). CFU counts (Figure 4D) were generally consistent with previous trends in assayed bacterial viability.

For both two pathogens, CFU counts were significantly lower in the ZIF-8 NPs group, with a 3-fold reduction in the *E. coli* group and a 6-fold reduction in *S. aureus* relative to the control group (*p* < 0.001) (Figures 4E,F). Rutin was clearly less effective as an antibacterial agent than the ZIF-8 group compared to the control group, but was still statistically different. Importantly, although ZIF-8@Rutin NPs had inferior antibacterial performance compared to ZIF-8 NPs, their CFU counts were still significantly different. The antibacterial performance of ZIF-8 NPs was minor diminished by the Rutin loading, but the novel ZIF-8@Rutin NPs still had sufficient bactericidal capacity.

The integrity of bacterial cell membranes is an essential indicator of the survival of bacteria. Finally, the structural alteration of the two bacteria was visualized by TEM analysis. As shown in Figures 4G,H, normal *S. aureus* and *E. coli* had intact and smooth cell membranes. In contrast, bacterial membranes treated with ZIF-8 NPs and ZIF-8@Rutin NPs collapsed. They underwent varying degrees of degradation, which is fatal for Gram-negative bacteria derived from the disruption of the outer membrane (Ruhal and Kataria, 2021). The TEM results indicated that one of the bactericidal mechanisms of ZIF-8@Rutin NPs is the disruption of the bacterial cell wall and cell membrane. Despite Gram-negative bacteria and Gram-positive bacteria differ in the cell wall structure, both pathogens showed almost identical susceptibility to the antibacterial function of ZIF-8 NPs and ZIF-8@Rutin NPs.

MOFs in antimicrobial applications could be segmented into two aspects according to their characteristics, namely metal ions and organic linkers. Some authors have indicated that the metal ions are possessed with positive charged, which could enhance the compatibility and the permeability of MOFs with the bacterial cell membrane (Chang et al., 2010; Li et al., 2021). Furthermore, the electrostatic gravitational effect could allow targeting on the bacterial surface (Yuan and Zhang, 2017; Yuan et al., 2019). The conjunction of these twin effects can effectively increase antimicrobial efficiency. Alternatively, the subversive ROS generated by Zn^2+^ could achieve a bactericidal effect by destroying the synthesis of bacterial biofilms and nucleic acid metabolism (Raghupathi et al., 2011; Li et al., 2012; Li et al., 2019b). Regarding the organic linker of ZIF-8 NPs, the imidazole ring has been verified to suppress bacterial growth, especially for the Gram-positive bacteria containing free fatty acids (Li et al., 2019a; Rashamuse et al., 2020).

The incorporation of Rutin reduced the antibacterial activity of ZIF-8 NPs in the present study, but Rutin has also been quoted as having definite antibacterial properties. A study demonstrated that the growth of *E. coli* was moderately restrained by Rutin (Wang et al., 2021). A further explanation is that Zn^2+^ toxicity is impaired under Rutin-rich conditions. Earlier studies have convincingly demonstrated that a dramatic decrease in extracellular ROS content occurs with increased Rutin, which reduces the effect of ROS on bacteria. Although relatively little research has been done on the antibacterial mechanism of flavonoids, flavonoids could function as an activator and synergistically in antimicrobial therapy (Amin et al., 2015; Deepika et al., 2019). In short, ZIF-8@Rutin NPs exhibited excellent antibacterial properties and opened up a new direction for clinical antibacterial applications.

### 3.4 Anti-inflammatory activity of ZIF-8@Rutin NPs

**FIGURE 5:**
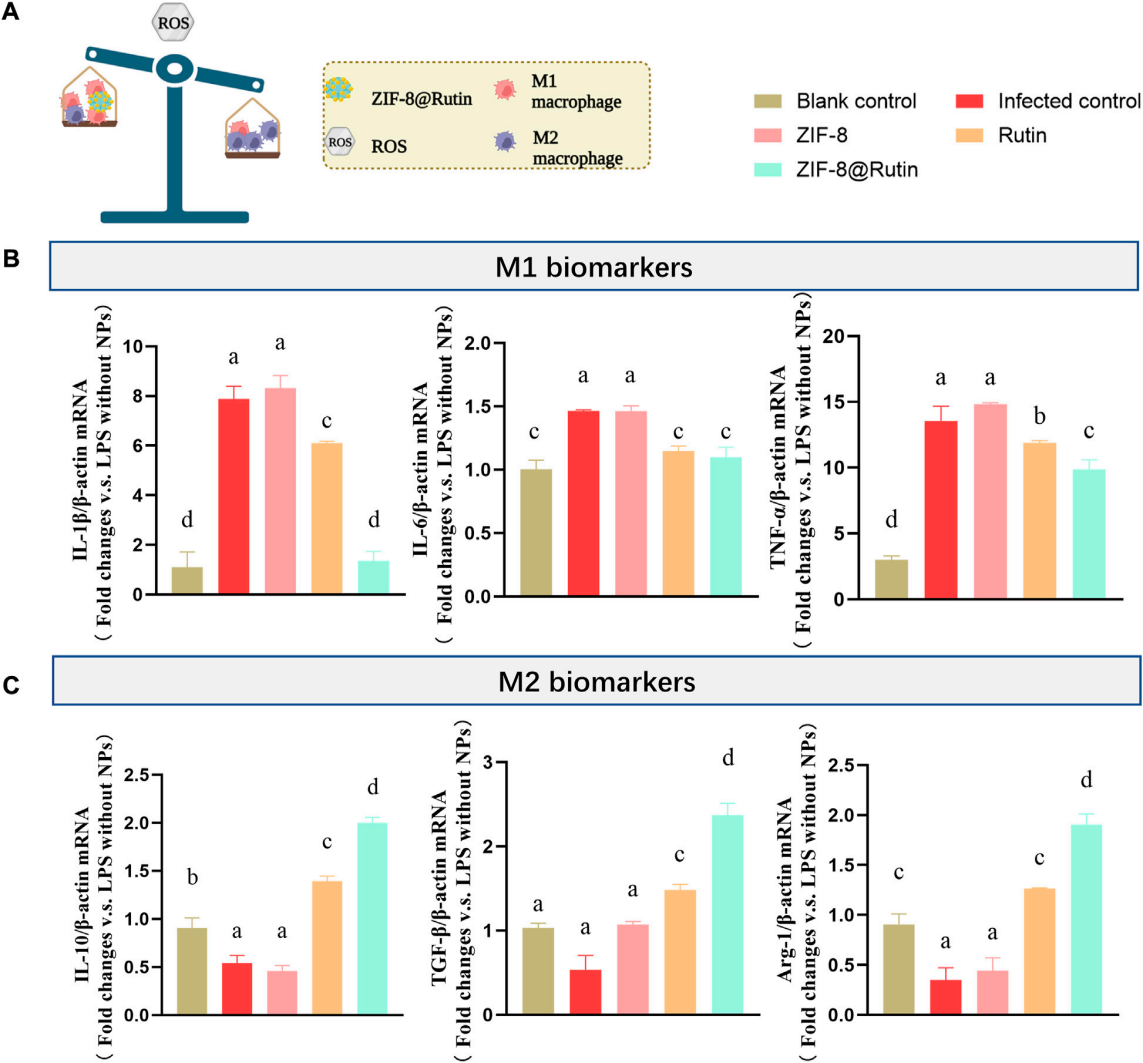
Schematic representation of the effect of nanocomposites on induced macrophage polarization. **(A)** Schematic representation of inflammation regulation by ZIF-8@Rutin NPs. **(B)**mRNA expression of pro-inflammatory cytokines (IL-1β, IL-6 and TNF-α) and **(C)** mRNA expression of anti-inflammatory cytokines (IL-10, TGF-β and Arg-1) after 3 h treatment with LPS (1 μg ml^−1^) and 24 h co-culture with ZIF-8 (30 μg ml^−1^), Rutin (40 μg ml^−1^), ZIF-8@Rutin (40 μg ml^−1^), respectively. (Values for blank group calibrated to 1. *n* = 3; mean ± sd; In each graph, the values of different letters differ significantly from each other).

Wound healing can be divided into four stages: hemostasis, inflammation, proliferation and maturation (Galiano et al., 2004; Guo and Dipietro, 2010). When the recovery of the wound enters the inflammatory phase, macrophages could polarize into classically activated macrophages (M1-type) and alternatively activated macrophages (M2-type). However, the first two polarities are almost opposite (Funes et al., 2018; Locati et al., 2020). The deterioration of local inflammation is a pathological manifestation of the macrophage polarization toward M1-type (pro-inflammatory) (Mosser and Edwards, 2008). When a microbial pathogen invades, M1-type macrophages mediate the first barrier by ROS. However, the function of ROS is non-targeted and equally aggressive towards normal cells, preventing tissue regeneration and wound healing (Shapouri-Moghaddam et al., 2018). Notably, the excessive pro-inflammatory capacity of M1-type can enhance the inflammatory response or promote the development of some inflammatory diseases. It has been reported that the high production of pro-inflammatory mediators (IL-1β, IL-6, and TNF-α) by M1-type macrophages exacerbated ischemia-reperfusion kidney injury (Lee et al., 2011). Nevertheless, the function of immune cells on macrophage polarization towards the M2-type (anti-inflammatory) is rather limited during the inflammatory repair phase. In comparison, M2 macrophages express arginase-1 (Arg-1) and IL-10, which exert anti-inflammatory, tissue repair and angiogenic effects (Wang et al., 2014). During the early stages of wound healing, the polarity of M1 macrophages and the subsequent release of inflammatory factors may be pivotal factors in causing inflammatory damage. According to the mechanism of the inflammatory response, the regulation of ROS is a focus for promoting the transformation of the M2 phenotype in macrophages (Figure 5A).

It is well established that macrophages are polarized to classically activated macrophages under the stimulation of lipopolysaccharide (LPS) (Mosser and Edwards, 2008). Thus, the positive control group adopted LPS to mimic bacterial invasion and produce an inflammatory response. Figure 5B demonstrated that ZIF-8@Rutin NPs significantly reduced the mRNA expression of M1-type macrophage related cytokines IL-1β, IL-6, and TNF-α compared to all other groups. Interestingly, as illustrated in Figure 5C, Rutin increased the mRNA expression of IL-10, TGF-β and Arg-1 associated with M2-type macrophages. Especially, ZIF-8@Rutin NPs showed the most substantial effect on the secretion of M2-type anti-inflammatory cytokines. The results revealed that the incorporation of Rutin exerted a definite anti-inflammatory effect and a potential to induce M2-type polarization of macrophages.

In response to this study, the introduction of the antioxidant drug Rutin into ZIF-8 NPs has achieved synergistic and enhanced modulation of local inflammatory immunity. Firstly, by virtue of its excellent antioxidant activity (as mentioned earlier in Figures 2D–F), Rutin could scavenge excessive ROS released by Zn^2+^ and reduce the oxidative stress damaging to tissues. In addition, Rutin has anti-inflammatory properties, mainly in balancing macrophage polarization. Previous studies have shown that quercetin which is in the same category as Rutin, could inhibit LPS-induced M1-type polarization, thereby reducing the release of inflammatory factors to cure kidney injury and fibrosis (Lu et al., 2018). It has also been suggested that the anti-inflammatory effect of Rutin is partly reflected in the protective effect on mitochondria, where mitochondrial dysfunction is an inducer of subsequent oxidative stress at the cellular and tissue level (Carrasco-Pozo et al., 2012). In brief, ZIF-8@Rutin NPs reversed the LPS-induced inflammatory responses by inducing macrophage polarization from M1 to M2 phenotypes and their antioxidant capacity.

### 3.5 Wound healing of infected trauma *in vivo*

**FIGURE 6:**
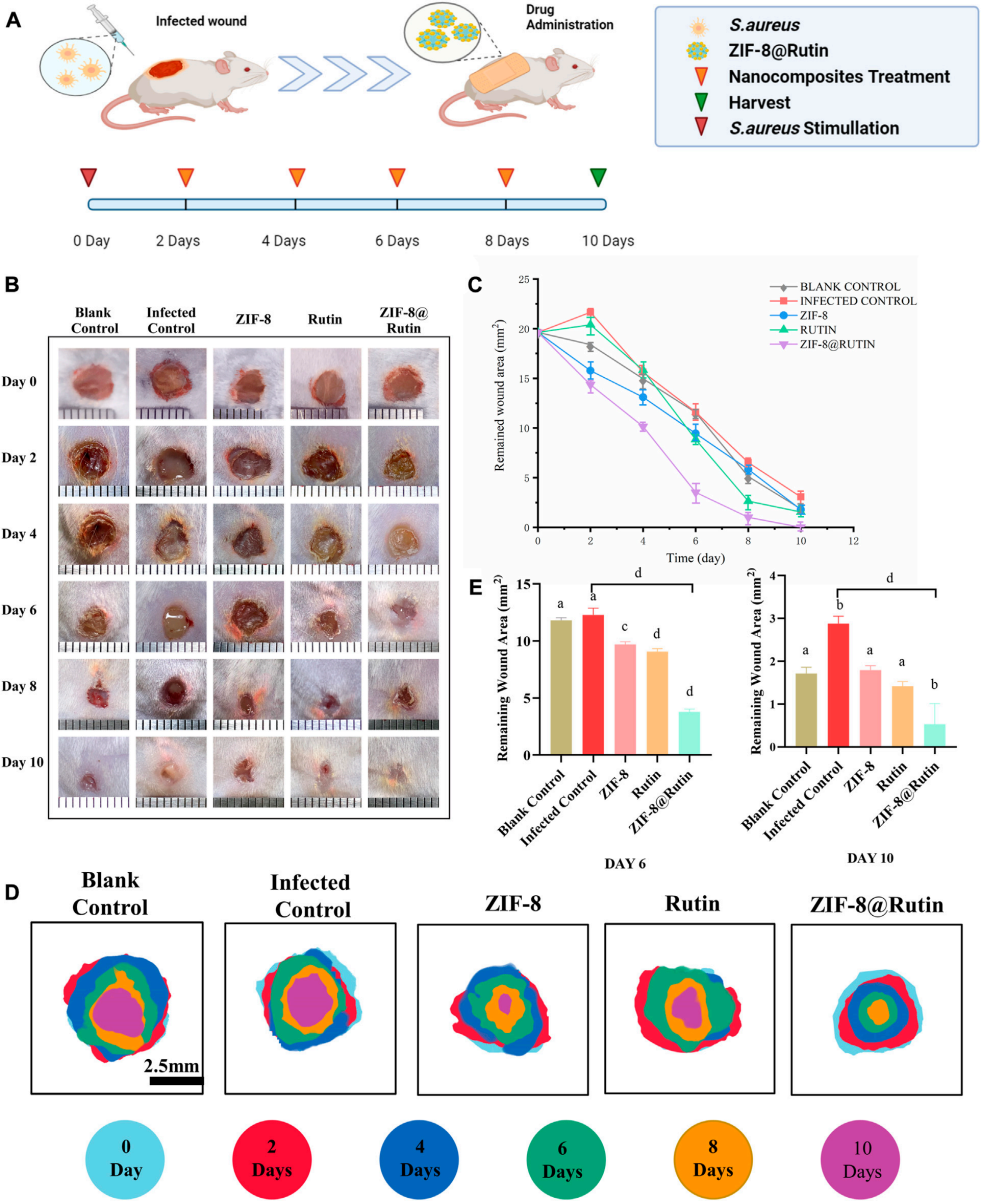
Facilitation of localized infected wound healing by ZIF-8@Rutin NPs *in vivo*. **(A)** Schematic diagram of the inflammation model on the dorsal skin of mice and the treatment process of ZIF-8@Rutin NPs. **(B)** Representative photographs of skin wounds at various stages of treatment with different dressings and **(C)** relative wound healing remaining area. **(D)** Schematic diagram of wound healing progression at different times. Blank controls were added with the same amount of PBS only, and PBS without NPs was added to wounds stimulated by *S. aureus* as infection control. **(E)** The remaining area of wound healing in each group on days 6 and 10. (Values for blank group calibrated to 1. *n* = 3; mean ± sd; In each graph, the values of different letters differed significantly from each other).

After verifying that ZIF-8@Rutin NPs have certain antibacterial and anti-inflammatory effects *in vitro*, we established an inflammation model in the back of mice to further explore the properties of ZIF-8@Rutin NPs. Figure 5A illustrates the process of establishing the *in vitro* model of inflammation previously described herein. Figure 6B was the representative image of the wounds in different experimental groups at 0, 2, 4, 6, 8, and 10 days. It can be visually observed that the infected control group had obvious pus-exudation within 10 days. Compared with the scars in the blank control group, the wounds in the experimental group tended to promote healing, and ZIF-8@Rutin NPs inhibited the occurrence of infection compared with the infection control group. Figure 6D was the residual area of the wound recorded at different stages in the whole *in vivo* experiment by image mapping. On the 6th day, the wound healing rate of the blank control group and the infected control group was approximately 41%, and the wound healing rate of the ZIF-8 NPs and Rutin groups was 52% and 55% (*p* < 0.001). Meanwhile, the wound healing rate of ZIF-8@Rutin NPs treated mice has been as high as 83% (*p* < 0.0001). On the 10th day, the wounds of ZIF-8@Rutin NPs treated mice had basically healed, while the wound healing rates of the blank and infected controls were approximately 91% and 85% (*p* < 0.001). The above figures (Figures 6C,E) indicated that ZIF-8@Rutin NPs boosted the healing rate of skin wounds in mice.

**FIGURE 7:**
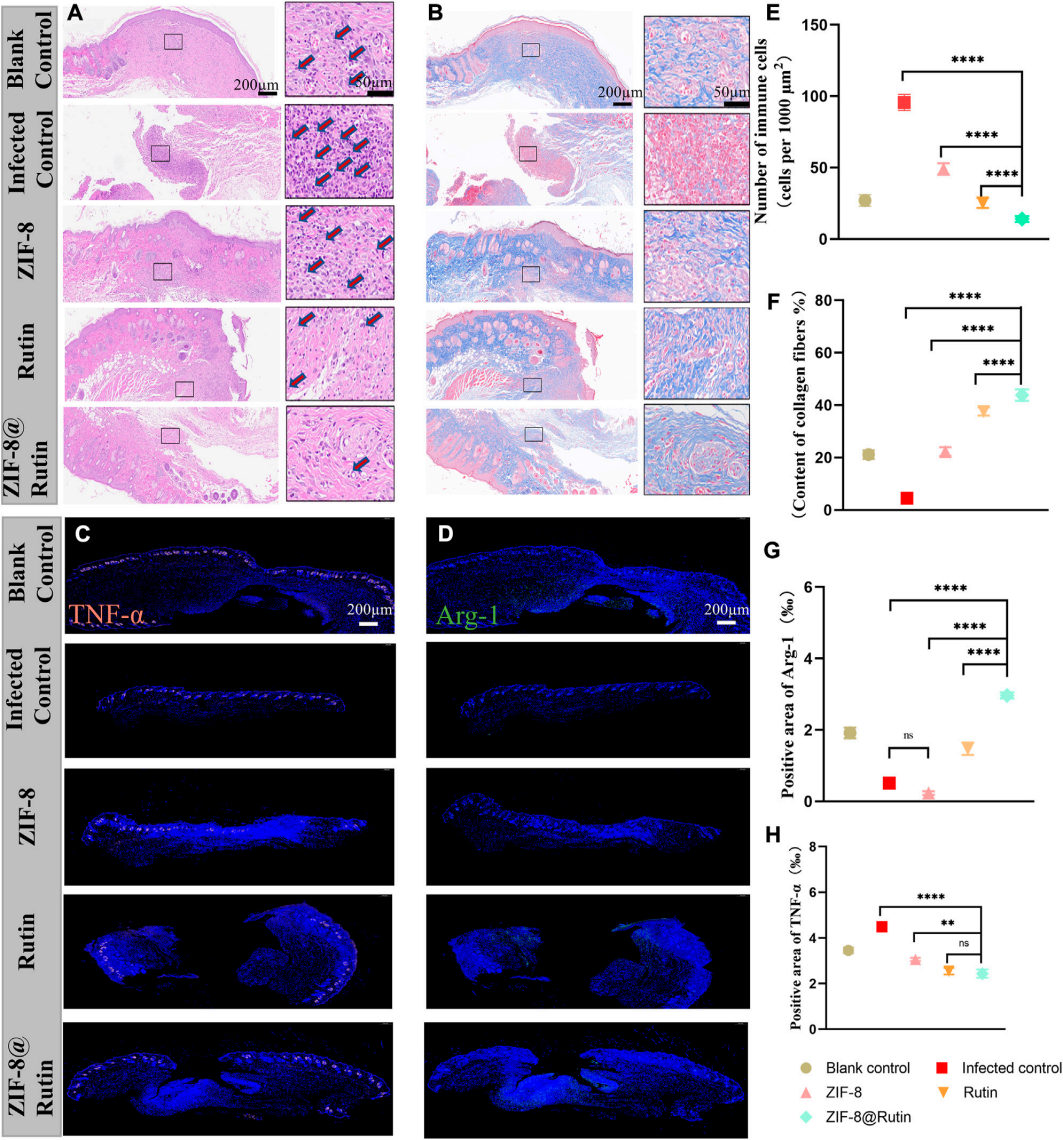
Histological evaluation of cutaneous wounds on the back of mice treated with different nanoparticle simulations. H&E **(A)** and Masson’s trichrome staining **(B)** of local peri-wound tissue on day 10. Immunofluorescence images of TNF-α **(C)** and Arg-1 **(D)** expression in wounds. In **(C,D)**, immunofluorescence staining (red) showed TNF-α-positive cells, and immunofluorescence staining (green) showed Arg-1-positive cells and DAPI staining (blue). Quantitative analysis of inflammatory cells in H&E samples from different nanocomposite treatment groups **(E)** and collagen fibrils in Masson’s samples **(F)**. Immunofluorescence intensity quantification of Arg-1 **(G)** and TNF-α **(H)**. (*n* = 6, **:*p* < 0.01; ****:*p* < 0.0001; ns: not significant.)

The histological assessment of the wounds on the 10th day was illustrated in Figure 7. H&E staining (Figure 7A) evidenced that the Rutin and ZIF-8@Rutin NPs groups had the least inflammatory cells (including neutrophils, macrophages, plasma cells, etc.), and the ZIF-8@Rutin NPs group had the best wound re-epithelialization (Figures 7B,F). The mild inflammatory response was (23.3 ± 0.16)/1000 μm^2^ in the blank control group and significantly (98.4 ± 0.98)/1000 μm^2^ in the infected control group. However, the ZIF-8 NPs, Rutin and ZIF-8@Rutin NPs groups were (49.51 ± 0.52), (24.1 ± 0.38) and (14.32 ± 0.63) respectively (Figure 7E).

ZIF-8@Rutin NPs had both the pathogen-killing function and the property of anti-inflammatory. Consequently, the skin wounds in the ZIF-8@Rutin NPs group exhibited the least inflammatory response and the fastest wound healing. The proliferative phase of wound healing is centred on fibroblasts, which produce large amounts of collagen and extracellular matrix, in concert with the rapid growth of endothelial cells (Han and Ceilley, 2017). The proliferative phase from the inflammatory phase to the healing process is characterized by the production of large amounts of collagen. Figure 7F demonstrated that plentiful collagen was particularly accumulated in the ZIF-8@Rutin NPs group and even far exceeded that of the blank one. The histomorphology features around the wound further validate the pharmacological efficacy of ZIF-8@Rutin NPs.

Furthermore, exposure to inflammation-associated cytokines in skin tissue was analysed by immunohistochemical staining and immunofluorescence intensity, which mechanistically demonstrated that ZIF-8@Rutin NPs have an anti-inflammatory effect of accelerating healing in infected wounds. Figures 7C,D shows that the production of TNF-α (red fluorescence) was markedly lower in the ZIF-8@Rutin NPs group compared to the infected control group, whereas the expression of Arg-1 (green fluorescence) was significantly higher. Apparently, the Rutin-doped ZIF-8 group significantly reduced TNF-α expression, increased Arg-1 expression compared to the ZIF-8 NPs group (Figures 7G,H). The same behavior of ZIF-8@Rutin NPs in inhibiting macrophage M1-type polarization and driving macrophage polarization towards the M2 phenotype was detected *in vitro* assays.

## 4 Conclusion

This study has shown a new type of multifunctional nanocomposite material (ZIF-8@Rutin NPs), which was developed by using the principle of electrostatic adsorption to aggregate the positively charged Rutin to the deprotonated 2-MI anions. The structure of ZIF-8 NPs is unchanged by loading with Rutin, but serves to optimize the cytotoxicity of ZIF-8. Synthetic ZIF-8@Rutin NPs have a sustained release of Zn^2+^ and Rutin, so they had remarkable antibacterial properties for *E. coli* and *S. aureus*. Although Rutin doping slightly reduced the antibacterial effect, the reduction of CFU was still statistically significant. Moreover, ZIF-8@Rutin NPs have a stronger anti-inflammatory capacity and can suppress the expression of pro-inflammatory cytokines (IL-1, IL-6 and TNF-α). At the same time, ZIF-8@Rutin NPs induced macrophage polarization towards the anti-inflammatory M2-type, which was manifested by the increased secretion of IL-10 and Arg-1. *In vivo* experiments have further demonstrated that ZIF-8@Rutin NPs had the ability to kill bacteria, fight inflammation and boost the healing of infected wounds. Therefore, this study developed a novel strategy to promote the healing of infected wounds that possessed strong antibacterial functions and excellent anti-inflammatory efficacy.

## Data Availability

The original contributions presented in the study are included in the article/supplementary material, further inquiries can be directed to the corresponding authors.
